# Data in support of substrate flexibility of a mutated acyltransferase domain and implications for polyketide biosynthesis

**DOI:** 10.1016/j.dib.2015.09.052

**Published:** 2015-10-14

**Authors:** Stephan Klopries, Kenny Bravo-Rodriguez, Kyra R.M. Koopmans, Uschi Sundermann, Samir Yahiaoui, Julia Arens, Susanna Kushnir, Elsa Sanchez-Garcia, Frank Schulz

**Affiliations:** a Fakultät für Chemie und Biochemie, Organische Chemie 1, Ruhr-Universität Bochum, Universitätsstraße 150, 44780 Bochum, Germany; b Max-Planck-Institut für Kohlenforschung, Kaiser-Wilhelm-Platz 1, 45470 Mülheim an der Ruhr, Germany; c Dr. Fooke-Achterrath Laboratorien GmbH, Habichtweg 16, 41468 Neuss, Germany; d Université de Caen Basse-Normandie, Centre d’Etudes et de Recherche sur le Médicament de Normandie, F-14032 Caen, France

## Abstract

Enzyme-directed mutasynthesis is an emerging strategy for the targeted derivatization of natural products. Here, data on the synthesis of malonic acid derivatives for feeding studies in *Saccharopolyspora erythraea* , the mutagenesis of DEBS and bioanalytical data on the experimental investigation of studies on the biosynthetic pathway towards erythromycin are presented.

**Specifications table**

**Table:** 

Subject area	*Chemistry, Biology*
More specific subject area	*Natural Products Biosynthesis*
Type of data	*Image (NMR-spectra of malonic acid derivatives), text file, figure*
How data was acquired	*NMR (Varian Mercury 400), mass spectrometry (LTQ Orbitrap)*
Data format	*Analyzed*
Experimental factors	*Synthesis products were analyzed after chromatographic purification, biosynthesis products were solid-phase extracted from fermentation broth*
Experimental features	*Analogues of biosynthetic building blocks were chemically synthesized and supplied to mutated strains of S. erythraea. LC–MS analysis of fermentation products revealed the substrate specificity of a key enzyme in polyketide biosynthesis*
Data source location	*Bochum, Germany and Mülheim an der Ruhr, Germany*
Data accessibility	*The data are supplied with this article*

**Value of the data**

• The preparative synthesis and handling of biosynthetic building block analogs is described.
• Analytical data on synthesized compounds are shown.
• Data on the site-directed mutagenesis of 6-deoxyerythronolide B synthase (DEBS) in *S. erythraea* are presented.

## 1 Data, experimental design, materials and methods

**Fig. 1:**
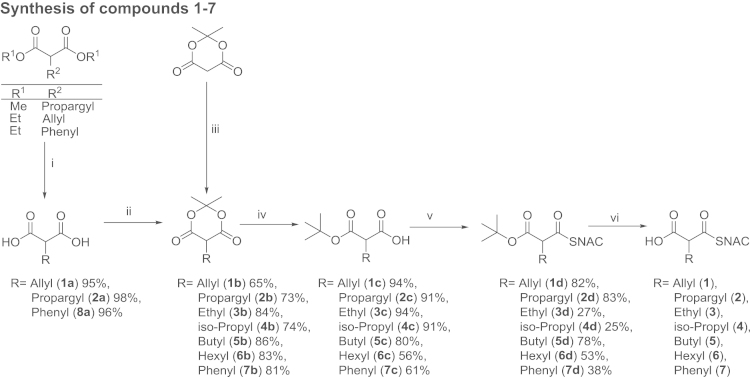
Synthesis of compounds **1–7**; (i) 3.0 eq. LiOH*H_2_O, H_2_O, 18 h, RT; (ii) 1.01 eq. isoprenylacetate, 0.06 eq. H_2_SO_4_, neat, 18 h, RT; (iii) 1.0 eq. boranedimethylamine complex, 3.0 eq. aldehyde or acetone, 1 h, RT; (iv) tBuOH, 6 h, 90–100 °C; (v) 1.1 eq. CDI, 0.3 eq. DMAP, 1.2 eq. SNAC, THF, 18 h, RT; (vi) 2.5 eq. TiCl_4_, DCM, 6 h, RT: room temperature, CDI: N,N′-Carbonyldiimidazole, DMAP: 4-Dimethylaminopyridine, SNAC: N-acetylcysteamine.

The data shown here substantiate the exploration of the mutated polyketide synthase which directs the biosynthesis of erythryomcin in *Saccharopolyspora erythraea*. In the sixth module of this polyketide synthase, the acyltransferase domain was recently mutated to accept propargylmalonyl-SNAC (**2**, Fig. 1) as substrate next to the native substrate methylmalonyl-CoA [1]. The critical mutation in the acyltransferase domain was V295A, located in the heart of the active center [2]. The mutation reduced the sterical hindrance on the substrate, allowing for the accommodation of **2**. Now, we explored the substrate flexibility of the DEBS AT6 V295A variant using a number of thioester-activated and differently substituted malonic acid derivatives. Biomolecular modeling was able to further the design and implementation of additional mutations in the active site of DEBS AT6, which decrease the steric constraints and improve the incorporation of the synthetic substrate **2** into the resulting polyketide. In this article, the synthesis of artificial extender unit analogs for polyketide biosynthesis and the mutagenesis of an acyltransferase domain for acceptance of these building blocks are described. Furthermore, the data on feeding experiments in *S. erythraea* are shown.

## 2 General information

Unless otherwise stated, materials for chemical synthesis were obtained from commercial suppliers (Sigma Aldrich, Alfa Aesar, Fluka, Acros) in the highest purity available and used without further purification. Solvents were dried following standard procedures [3]. Solvents used for extraction and chromatography were purchased from Thermo Fisher Scientific. Flash chromatography was carried out using Acros silica gel 60 (35–70 μm mesh). Thin-layer chromatography (TLC) was performed on aluminum-backed, precoated silica gel (60 F245) from Merck with cyclohexane/EtOAc or DCM/MeOH mixtures as mobile phases. Spots were detected by staining with KMnO_4_ solution (5.0 g KMnO_4_, 33 g K_2_CO_3_, 10 mL 5% aqueous NaOH in 500 mL H_2_O) and subsequent heat treatment.

NMR spectra were recorded by using a Varian Mercury 400 (400 MHz, ^1^H; 100 MHz, ^13^C) spectrometer and calibrated using residual undeuterated solvent as an internal reference. Data are shown in Supplementary File 1.

High-resolution mass spectra were recorded on a LTQ Orbitrap with Accela HPLC-System (column Hypersil Gold, length 50 mm, inside diameter 1 mm, particle size 1.9 μm, ionization method: Electrospray Ionization). Products were characterized by NMR (^1^H, ^13^C) and HRMS.

For mass spectrometric detection the electrospray ionization was carried out in positive ionization mode by using a source voltage of 4 kV. The capillary voltage was set to 18 V, the capillary temperature to 275 °C, and the tube lens voltage to 115 V. Spectra were acquired in full scan centroid mode with a mass-to-charge range from 200 to 2000.

## 3 Synthesis of compounds 1–7

**Synthesis of *N*-acetylcysteamine (SNAC)** [4]: 20.00 g (176 mmol) cysteamine hydrochloride, 11.62 g (259 mmol) KOH (85%) and 36.97 g (440 mmol) NaHCO_3_ were added to 500 mL of deionized H_2_O. After everything was dissolved, 19.77 g (18.31 ml, 259 mmol) acetic anhydride was added dropwise at 0 °C. After stirring at room temperature for 18 h, the light rose solution was brought to pH 1 with conc. HCl and the colorless solution was extracted three times with 150 ml EtOAc. The combined organic layers were dried over Na_2_SO_4_ to obtain 20.47 g (98%) of the desired product as colorless oil.

**^1^H NMR** (400 MHz, CDCl_3_-d_1_): 1.34–1.38 (t, *J*=8.4 Hz, 1H), 1.97 (s, 3H), 2.60–2.66 (m, 2H), 3.36–3.40 (m, 2H), 6.33 (bs, 1H); **^13^C NMR** (101 MHz, CDCl_3_-d_1_): 23.1, 24.5, 42.6, 170.5; **HRMS:** calc. for 120.04776 C_4_H_10_ONS [M+H]^+^; found: 120.04730 C_4_H_10_ONS [M+H]^+^; ***R*_f_:** 0.42 (DCM/MeOH 9:1, KMnO_4_).

**General procedure for the saponification of malonic acid diesters 1a, 2a+7a:** The commercially available malonic diester was added to H_2_O (10 ml/g) and 3.0 eq LiOH*H_2_O were added at once. The solution was stirred for 18 h, then washed with 100 ml Et_2_O. The aqueous phase was acidified to pH 1 using conc. HCl and extracted three times with 150 ml EtOAc. The combined organic layers were dried over Na_2_SO_4_ to obtain the desired product as white solid.

**2-Allyl-malonic acid (1a): ^1^H NMR:** (400 MHz, D_2_O-d_2_) δ=2.49–2.51 (m, 2H), 3.17–3.21 (t, *J*=7.8 Hz, 1H), 5.04–5.16 (m, 2H), 5.83–5.93 (m, 1H); **^13^**C **NMR:** (101 MHz, MeOD-d_4_) δ=34.1, 52.9, 117.5, 135.8; 172.5; **mp:** 103.3–103.6 °C; yield: 18.59 g; 95% (27.0 g scale, 134.8 mmol).

**2-(Prop-2-yn-1-yl)malonic acid (2a)**: **^1^H NMR:** (400 MHz, MeOD-d_4_) δ=2.31–2.32 (t, *J*=2.7 Hz, 1H), 2.68–2.71 (dd, *J*=7.6, 2.7 Hz, 2H), 3.49–3.53 (t, *J*=7.6 Hz, 1H, 2-H, CH); **^13^**C NMR: (101 MHz, MeOD-d_4_) δ=19.2, 52.6, 71.2, 81.4, 171.4; **mp:** 141–141.6 °C; yield: 10.42 g; 98% (12.6 g scale, 74.13 mmol).

**2-Phenylmalonic acid (7a): ^1^H NMR** : (400 MHz, MeOD-d_4_) δ=4.65 (s, 1H), 4.88 (bs, 2H), 7.30–7.37 (m, 3H), 7.39–7.42 (m, 2H); **^13^C NMR:** (101 MHz, MeOD-d_4_) δ=59.0, 128.9, 129.4, 130.3, 130.4, 135.2, 171.9; **HRMS:** calc.: 179.03498 C_9_H_7_O_4_ [M−H]; found: 179.03568 C_9_H_7_O_4_ [M−H]; **mp:** 160.4-160.5 °C; yield: 7.29 g; 96% (10.0 g scale, 42.33 mmol).

**General procedure for the synthesis of Meldrum׳s acid derivatives 1b, 2b+7b** [5]:

For the formation of Meldrum׳s acid derivatives **1b, 2b+8b** the general procedure of Singh and Danishefsky was used [5]. 1.01 eq. isoprenylacetate was added under argon protection to the corresponding malonic acid derivative. To the resulting white slurry 0.06 eq. sulfuric acid were added dropwise at 0 °C. The resulting yellow to brown solution was stirred for 18 h to reach room temperature. 100 g ice and 10 ml 1 M HCl were added to the brown reaction mixture (at 10 g synthesis scale). The resulting precipitate was filtered and washed twice with 20 ml cold water.

In cases where the reaction mixture became solid after 18 h, water was added to form a slurry. To this slurry 100 g ice and 10 ml 1 M HCl were added (for 10 g synthesis scale). The resulting precipitate was filtered and washed twice with 20 ml of ice-cold water. The resulting white to brown product usually was directly submitted to the next synthesis step.

If material of higher purity was needed the white to brown solids obtained from the first precipitation were dissolved in a small volume MeOH at RT. After adding ice and a few drops of conc. HCl the white precipitate was filtered and washed twice with 20 ml of ice cold water.

**5-Allyl-2,2-dimethyl-1,3-dioxane-4,6-dione (1b): ^1^H NMR:** (400 MHz, CDCl_3_-d_1_) δ=1.76 (s, 3H), 1.79 (s, 3H), 2.86–2.90 (m, 2H), 3.57–3.60 (t, *J*=5.3Hz, 1H), 5.14–5.26 (m, 2H), 5.81–5.92 (m, 1H); **^13^C NMR:** (101 MHz, CDCl_3_-d_1_) δ=27.2, 28.6, 30.5, 46.4, 105.1, 132.8, 165.1; **HRMS:** calc.: 185.08084 C_9_H_13_O_4_ [M+H]^+^;found: 185.08071 C_9_H_13_O_4_ [M+H]^+^; **mp:** 71 °C; ***R*_f_:** 0.56 (EtOAc/cyclohexane 1:1, KMnO_4_); yield: 23.05 g; 65% (27.95 g scale, 193.96 mmol).

**2,2-Dimethyl-5-(prop-2-yn-1-yl)-1,3-dioxane-4,6-dione (2b): ^1^H NMR:** (400 MHz, CDCl_3_-d_1_) δ=1.80 (s, 3H), 1.81 (s, 3H), 2.05–2.06 (t, *J*=2.6 Hz, 1H), 3.02–3.04 (dd, *J*=4.9, 2.6 Hz, 2H), 3.67–3.96 (t, *J*=4.9 Hz, 1H); **^13^C NMR:** (101 MHz, CDCl_3_-d_1_) δ=16.7, 27.2, 28.7, 46.1, 70.9, 79.4, 105.5, 164.1; **HRMS:** calc.: 183.06519 C_9_H_11_O_4_ [M+H]^+^; found: 183.06512 C_9_H_11_O_4_ [M+H]^+^; **mp:** 140.0–140.4 °C; ***R*_f_:** 0.66 (EtOAc/cyclohexane 1:1, KMnO_4_); yield: 29.67 g; 73% (31.9 g scale, 224.47 mmol).

2,2-Dimethyl-5-phenyl-1,3-dioxane-4,6-dione (7b): **^1^H NMR:** (400 MHz, CDCl_3_-d_1_) δ=1.75 (s, 3H), 1.87 (s, 3H), 4.77 (s, 1H), 7.28–7.31 (m, 2H), 7.37–7.45 (m, 3H); **^13^C NMR:** (101 MHz, CDCl_3_-d_1_) δ=27.7, 28.7, 52.9, 105.8, 128.9, 129.2, 129.3, 130.7, 164.8; **HRMS:** calc.: 221.08084 C_12_H_13_O_4_ [M+H]^+^; found: 221.08113 C_12_H_13_O_4_ [M+H]^+^; **mp:** 140.1–142.3 decomposition; ***R*_f_:** 0.18 (EtOAc/cyclohexane 1:1, KMnO_4_); yield: 5.96 g; 81% (6.0 g scale, 33.30 mmol).

**General procedure for the reductive alkylation of Meldrum׳s acid 3b–6b** [6]**:**

The alkylation was carried out as described by Hurubowchak and Smith [6]. Meldrum׳s acid was dissolved in abs. MeOH. Subsequently, 1.01 eq. boranedimethylamine complex were added. After the borane was dissolved completely 3.0 eq. of the corresponding aldehyde were added in 3 min at RT under a stream of N_2_. After 1 h the yellow reaction mixture was quenched by 100 g ice and 10 ml of 1 M HCl. The resulting suspension was filtered and washed twice with 25 ml cold water. The resulting white solid was dried *in vacuo* and can directly be submitted to the next reaction step.

**Synthesis of Isopropyl-meldrum׳s acid (4b):**

To 8 ml Acetone (freshly dried over 4 A°-molecular sieve), 4.0 g (27.75 mmol) meldrum׳s acid were added under argon atmosphere. At 0 °C 1.68 g (28.03 mmol) borane dimethylamine complex was added. After 15 min the ice bath was removed and the reaction mixture was stierred for 18 h at room temperature. The yellow solution was poured on 80 cm^3^ ice and acidified with 3 ml 1 N HCl. The resulting precipitate was filtered and washed twice with 20 ml ice cold H_2_O.

If material of higher purity was needed, the white to brown solids obtained from the first precipitation were dissolved in a minimum of MeOH at RT. After adding ice and a view drops of conc. HCl the white precipitate was filtered and washed twice with 20 ml of ice cold water.

**5-Ethyl-2,2-dimethyl-1,3-dioxane-4,6-dione (3b): ^1^H NMR:** (400 MHz, CDCl_3_-d_1_) δ=1.03–1.07 (t, *J*=7.3 Hz, 3H), 1.75 (s, 3H), 1.78 (s, 3H), 2.14–2.20 (qd, *J*=7.3, 4.9 Hz, 2H), 3.48–3.50 (t, *J*=4.9 Hz, 1H); **^13^C NMR:** (101 MHz, CDCl_3_-d_1_) δ=10.9, 20.3, 27.1, 28.6, 47.3, 104.9, 165.57; **HRMS:** calc.: 173.08084 C_8_H_13_O_4_ [M+H]^+^; found: 173.08065 C_8_H_13_O_4_ [M+H]^+^; **mp:** 110–110.2 °C; ***R*_f_:** 0.66 (EtOAc/cyclohexane 1:1, KMnO_4_); yield: 3.50 g; 84% (4.0 g scale, 27.75 mmol).

**5-Isopropyl-2,2-dimethyl-1,3-dioxane-4,6-dione (4b): ^1^H NMR:** (400 MHz, CDCl_3_-d_1_) δ=1.16 (s, 3H) 1.18 (s, 3H),1.73–1.74 (d, *J*=6.0 Hz, 6H), 2.73–2.78 (m, 1H), 3.37 (d, *J*=3.1 Hz, 1H); **^13^C NMR:** (101 MHz, CDCl_3_-d_1_) δ=19.3, 27.6, 28.4, 29.2, 51.8, 104.8, 165.1; **HRMS:** calc.: 187.09649 C_9_H_15_O_4_ [M+H]^+^; found: 187.09638 C_9_H_15_O_4_ [M+H]^+^; **mp:** 104 °C; ***R*****_f_:** 0.71 (1:1 EtOAc/cyclohexane, KMnO_4_); yield: 3.80 g; 74% (4.0 g scale, 27.75 mmol).

**5-Butyl-2,2-dimethyl-1,3-dioxane-4,6-dione (5b): ^1^H NMR:** (400 MHz, CDCl_3_-d_1_)δ=0.90–0.93 (t, *J*=7.1 Hz), 1.32–1.47 (m, 4H), 1.73 (s, 3H), 1.78 (s, 3H), 2.07-2.13 (m, 2H), 3.47–3.50 (t, *J*=5.1 Hz, 1H); **^13^**C NMR: (101 MHz, CDCl_3_-d_1_) δ=13.9, 22.8, 26.6, 27.1, 28.6, 28.8, 46.3, 104.9, 165.80; **HRMS:** calc.: 201.11214 C_10_H_17_O_4_ [M+H]^+^; found: 201.11206 C_10_H_17_O_4_ [M+H]^+^; **mp:** 55.6-56.1 °C; ***R*_f_:** 0.73 (EtOAc/cyclohexane 1:1, KMnO_4_); yield: 19.31 g; 86% (18.0 g scale, 123.17 mmol).

**5-Hexyl-2,2-dimethyl-1,3-dioxane-4,6-dione (6b): ^1^H NMR:** (400 MHz, CDCl_3_-d_1_) δ=0.85–0.89 (t, *J*=6.5 Hz), 1.28–1.35 (6H), 1.40–1.47 (2H), 1.75 (3H), 1.77 (3H), 2.06–2.12 (2H), 3.47–3.50 (t, *J*=5.0 Hz, 1H); **^13^C NMR:** (101 MHz, CDCl_3_-d_1_) δ=14.1, 22.6, 26.6, 26.9, 27.1, 28.6, 29.3, 31.56, 46.3, 104.9, 165.8; **HRMS:** calc.: 229.14344 C_12_H_21_O_4_ [M+H]^+^; found: 229.14332 C_12_H_21_O_4_ [M+H]^+^; ***R*_f_:** 0.50 (DCM/MeOH, KMnO_4_); yield: 13.12 g; 83% (10.0 g scale; 69.38 mmol).

**General procedure for the synthesis*t*Butylmalonic acids 1c–7c:**

*t*BuOH (125 ml/10 g) was added to Meldrum׳s acid and heated up to 95–100 °C for 6 h (DC-control). Then *t*BuOH was evaporated *in vacuo* and the resulting oil was purified by column chromatography (PE/EtOAc 1:0→ 85:15, gradient in 5%-steps) to obtain the desired products as clear oils.

**2-(*tert*-Butoxycarbonyl)pent-4-enoic acid (1c): ^1^H NMR:** (400 MHz, CDCl_3_-d_1_) δ=1.47 (s, 9H), 2.58–2.68 (m, 2H), 3.36–3.40 (t, *J*=7.3 Hz, 1H), 5.07–5.16 (m, 2H), 5.73–5.83 (m, 1H); **^13^C NMR:** (101 MHz, CDCl_3_-d_1_) δ=28.0, 33.2, 52.1, 82.8, 117.9, 133.8, 168.5, 174.4; **HRMS:** calc.: 201.11214 C_10_H_17_O_4_ [M+H]^+^, 223.09408 C_10_H_16_O_4_Na [M+Na]^+^,218.13868 C_10_H_20_O_4_N [M+NH_4_]^+^; found: 201.11217 C_10_H_17_O_4_ [M+H]^+^, 223.09421 C_10_H_16_O_4_Na [M+Na]^+^,218.13878 C_10_H_20_O_4_N [M+NH_4_]^+^; ***R*_f_:** 0.55 (EtOAc/cyclohexane, KMnO_4_); yield: 3.01 g; 91% (3.0 g scale, 35.34 mmol).

**2-(*tert*-Butoxycarbonyl)-pent-4-yl acid (2c): ^1^H NMR:** (400 MHz, CDCl_3_-d_1_) δ=1.46 (s, 9H), 2.04 (t, *J*=2.6 Hz, 1H), 2.17–2.18 (s, 1H), 2.94–2.95 (d, *J*=2.6 Hz, 2H); **^13^C NMR:** (101 MHz, CDCl_3_-d_1_) δ=23.1, 28.1, 57.2, 72.2, 78.8, 83.8, 167.7, 174.4; **HRMS:** calc.: 199.09649 C_10_H_15_O_4_ [M+H]^+^, 221.07843 C_10_H_15_O_4_Na [M+Na]^+^; found: 221.07845 C_10_H_15_O_4_, [M+Na]^+^; **mp:** 95.6–96.7 °C; ***R*_f_:** 0.54 (MeOH/CHCl_3_ 1:9, KMnO_4_); yield: 30.35 g; 94% (29.67 g scale, 162.8 mmol).

**2-(*tert*-Butoxycarbonyl)butanoicacid (3c): ^1^H NMR:** (400 MHz, CDCl_3_-d_1_) δ=0.97–1.01 (t, *J*=7.4 Hz, 3H), 1.48 (s, 9H), 1.89–1.96 (m, 2H), 3.20–3.24 (t, *J*=7.2 Hz, 1H); **^13^C NMR:** (101 MHz, CDCl_3_-d_1_) δ=11.8, 22.8, 28.0, 53.9, 82.7, 169.4, 174.7; **HRMS:** calc.: 187.09758 C_9_H_15_O_4_ [M−H]; found: 187.09826 C_9_H_15_O_4_ [M−H]; **mp:** 53.8-54.1 °C; ***R*_f_:** 0.0–0.65 (EtOAc/cyclohexane 1:1, KMnO_4_); yield: 7.38 g; 94% (7.17 g scale, 41.64 mmol).

**2-(*tert*-Butoxycarbonyl)-3-methylbutanoic acid (4c): ^1^H NMR:** 1.03–1.04 (d, *J*=2.2 Hz), 1.05 (d, *J*=2.2 Hz), 1.48 (s, 9H), 2.29–2.40 (m, 1H), 3.08–3.10 (d, *J*=7.7 Hz, 1H); **^13^C NMR:** (101 MHz, CDCl_3_-d_1_) δ=20.3, 20.5, 28.1, 29.8, 59.2, 82.9, 169.4, 173.5; **HRMS:** calc: 201.11323 C_10_H_17_O_4_ [M−H]; found: 201.11372 C_10_H_17_O_4_ [M−H]; **mp:** 64.3–65.2 °C; ***R*_f_:** 0.08-0.55 (EtOAc/cyclohexane 1:1, KMnO_4_); yield: 5.07 g; 91% (5.1 g scale, 27.3 mmol).

**2-(*tert*-Butoxycarbonyl)hexanoicacid (5c): ^1^H NMR:** (400 MHz, CDCl_3_-d_1_) δ=0.88–0.92 (t, *J*=7.0 Hz, 3H), 1.32–1.35 (m, 4H), 1.47 (s, 9H), 1.85–1.91 (m, 2H), 3.25–3.29 (t, *J*=7.4 Hz, 1H); **^13^C NMR:** (101 MHz, CDCl_3_-d_1_) δ=13.9, 22.5, 28.0, 28.9, 29.5, 52.5, 82.6, 164.3, 174.9; **HRMS:** calc.: 215.12888 C_11_H_19_O_4_ [M−H]; found: 215.12939 C_11_H_19_O_4_ [M−H]; ***R*_f_:** 0–0.51 (EtOAc/cyclohexane 1:1, KMnO_4_); yield: 2.73 g; 80% (2.8 g scale, 14.0 mmol).

**2-(*tert*-Butoxycarbonyl)octanoicacid (6c): ^1^H NMR:** (400 MHz, CDCl_3_-d_1_) δ=0.86–0.89 (t, *J*=6.9 Hz, 3H), 1.28–1.32 (m, 8H), 1.47 (s, 9H), 1.84–1.91 (m, 2H), 3.26–3.29 (t, *J*=7.3 Hz, 3H); **^13^C NMR:** (101 MHz, CDCl_3_-d_1_) δ=14.2, 22.6, 27.3, 28.0, 28.9, 29.4, 31.6, 52.5, 82.7, 169.4, 175.1; **HRMS:** calc.: 243.16018 C_13_H_23_O_4_ [M−H]; found: 243.16086 C_13_H_23_O_4_ [M−H]; ***R*_f_**: 0–0.47 (EtOAc/cyclohexane 1:1, KMnO_4_); yield: 8.02 g; 56% (13.2 g scale, 57.8 mmol).

**3-(*tert*-Butoxy)-3-oxo-2-phenylpropanoic acid (7c): ^1^H NMR:** (400 MHz, CDCl_3_-d_1_) δ=1.44 (s, 9H), 4.55 (s, 1H), 7.28–7.40 (m, 5H); **^13^C NMR:** (101 MHz, CDCl_3_-d_1_) δ=27.9, 58.3, 83.5, 128.5, 128.9, 129.1, 132.8, 168.0, 173.1; **HRMS:** calc.: 237.11214 C_13_H_16_O_4_ [M+H]^+^; found: 191.10828 C_12_H_16_O_2_ [M-CO_2_]; **mp:** 100.9–101.3 °C; ***R*_f_:** 0.0–0.50 (EtOAc/cyclohexane 1:1, KMnO_4_); yield: 3.26 g; 61% (5.0 g scale, 22.7 mmol).

**General procedure for the thioesterfication of compounds 1d–7d:**

*tert*-Butylcarboxylic acid was dissolved in abs. THF (10 ml/g) under argon. Subsequently, 1.2 eq. CDI was added at 0 °C, and the mixture was stirred for 30 min at 0 °C followed by 3 h at RT before 0.3 eq. DMAP and 1.3 eq. SNAC were added. After 18 h at RT the solvent was removed *in vacuo* and the residue was suspended 300 ml EtOAc and washed three times with 100 ml 1 M K_2_CO_3_ and twice with 100 ml 1 M HCl. The organic layer was dried over Na_2_SO_4_, and purified by column chromatography (DCM/MeOH 99:1) to obtain the desired thioesters as slightly yellow oils.

***tert*-Butyl 2-(((2-acetamidoethyl)thio)carbonyl)pent-4-enoate (1d): ^1^H NMR:** (400 MHz, CDCl_3_-d_1_) δ=1.43 (s, 9H), 1.94 (s, 3H), 2.58–2.62 (m, 2H), 2.99–3.11 (m, 2H), 3.34–3.48 (m, 2H), 3.55–3.59 (t, *J*=7.5 Hz, 1H), 5.03–5.12 (m, 2H), 5.66–5.77 (m, 1H), 6.00 (bs, 1H); **^13^C NMR:** (101 MHz, CDCl_3_-d_1_) δ=23.3, 28.1, 28.9, 33.5, 39.6, 60.6, 82.7, 117.9, 133.9, 167.4, 170.6, 195.5; **HRMS:** calc: 302.14206 C_14_H_24_O_4_NS[M+H]^+^, 324.12400 C_14_H_23_O_4_NNaS [M+Na]^+^, 319.16860 C_14_H_27_O_4_N_2_S [M+NH_4_]^+^; found: 302.14231 C_14_H_24_O_4_NS[M+H]^+^, 324.12418 C_14_H_23_O_4_NNaS [M+Na]^+^, 319.16919 C_14_H_27_O_4_N_2_S [M+NH_4_]^+^; ***R*_f_**: 0.68 (DCM/MeOH 9:1, KMnO_4_); yield: 3.73 g; 82% (3.02 g scale, 15.06 mmol).

***tert-*Butyl 2-(((2-acetamidoethyl)thio)-carbonyl)pent-4-ynoate (2d): ^1^H NMR:** (400 MHz, CDCl_3_-d_1_) δ=1.47 (s, 9H),1.96 (s, 3H),2.01–3.03 (t, *J*=2.7 Hz, 1H),2.74–2.76(dd, *J*=7.6, 2.7,0.6 Hz, 2H),3.09-3.12 (m, 2H),3.43-3.47 (m, 2H), 3.69-3.73 (t, *J*=7.6 Hz, 1H),5.87 (bs, 1H); **^13^C NMR:** (101 MHz, CDCl_3_-d_1_)δ=18.8, 23.3, 27.9, 29.1, 39.5, 59.6, 70.7, 79.9, 83.3, 166.3, 170.5, 194.4; **HRMS:** calc.: 300.12641 C_14_H_22_O_4_NS [M+H]^+^, 322.10835 C_14_H_22_O_4_NSNa [M+Na]^+^, 317.15295 C_14_H_25_O_4_N_2_S [M+NH_4_]^+^; found: 300.12664 C_14_H_22_O_4_NS [M+H]^+^, 322.10861 C_14_H_22_O_4_NSNa [M+Na]^+^, 317.15325 C_14_H_25_O_4_N_2_S,[M+NH_4_]^+^; ***R*_f_**: 0.69 (DCM/MeOH 9:1, KMnO_4_); yield: 83% (10.8 g scale, 54.49 mmol).

***tert*-Butyl 2-(((2-acetamidoethyl)thio)carbonyl)butanoate (3d): ^1^H NMR:** (400 MHz, CDCl_3_-d_1_) δ=0.90–0.94 (t, *J*=7.4 Hz, 3H), 1.43 (s, 9H), 1.84–1.91 (m, 2H), 1.93 (s, 3H), 2.98–3.10 (m, 2H), 3.34–3.46 (m, 3H), 6.09 (bs, 1H); **^13^C NMR:** (101 MHz, CDCl_3_-d_1_) δ=11.8, 22.9, 23.2, 27.9, 28.7, 39.6, 62.7, 82.3, 167.8, 170.5, 196.0; **HRMS:** calc.: 290.14206 C_13_H_24_O_4_NS[M+H]^+^, 312.12400 C_13_H_23_O_4_NNaS [M+Na]^+^, 307.16860 C_13_H_27_O_4_N_2_S [M+NH_4_]^+^; found: 290.14246 C_13_H_24_O_4_NS[M+H]^+^, 312.12437 C_13_H_23_O_4_NNaS [M+Na]^+^, 307.16939 C_13_H_27_O_4_N_2_S [M+NH_4_]^+^; ***R*_f_**: 0.67 (DCM/MeOH 9:1, KMnO_4_); yield: 786 mg; 27% (1.91 g scale, 10.13 mmol).

***tert*-Butyl 2-(((2-acetamidoethyl)thio)carbonyl)-3-methylbutanoate (4d): ^1^H NMR:** (400 MHz, CDCl_3_-d_1_) δ=0.94–0.96 (d, *J*=6.7 Hz, 3H), 0.98–0.99 (d, *J*=6.7 Hz, 3H), 1.43 (s, 9H), 1.95 (s, 3H), 2.45–2.39 (m, 1H), 2.99–3.13 (m, 2H), 3.23–3.25 (d, *J*=9.5 Hz, 1H), 3.36–3.50 (m, 2H), 5.87 (bs, 1H); **^13^C NMR:** (101 MHz, CDCl_3_-d_1_) δ=20.5, 20.6, 23.5, 28.2, 28.9, 30.0, 39.9, 68.9, 82.5, 167.4, 170.6, 195.6; **HRMS:** calc.: 304.15771 C_14_H_26_O_4_NS[M+H]^+^, 326.13965 C_14_H_25_O_4_NNaS [M+Na]^+^, 321.18425 C_14_H_29_O_4_N_2_S [M+NH_4_]^+^; found: 304.15825 C_14_H_26_O_4_NS[M+H]^+^, 326.14008 C_14_H_25_O_4_NNaS [M+Na]^+^, 321.18516 C_14_H_29_O_4_N_2_S [M+NH_4_]^+^; ***R*_f_**: 0.64 (DCM/MeOH 9:1, KMnO_4_); yield: 598 mg; 25% (1.58 g scale, 7.81 mmol).

***tert*-Butyl 2-(((2-acetamidoethyl)thio)carbonyl)hexanoate (5d): ^1^H NMR:** (400 MHz, CDCl_3_-d_1_) δ=0.84–0.88 (t, *J*=7.0 Hz, 3H), 1.26–1.28 (m, 4H), 1.42 (s, 9H), 1.80–1.87 (m, 2H), 1.96 (s, 3H), 2.99–3.08 (m, 2H), 3.33–3.47 (m, 3H), 6.09 (bs, 1H); **^13^C NMR:** (101 MHz, CDCl_3_-d_1_) δ=13.8, 22.4, 23.2, 27.9, 28.7, 29.2, 29.4, 39.6, 61.2, 82.3, 167.9, 170.5, 196.1; **HRMS:** calc.: 318.17336 C_15_H_28_O_4_NS [M+H]^+^, 340.15530 C_15_H_27_O_4_NNaS [M+Na]^+^, 335.19990 C_15_H_31_O_4_N_2_S [M+NH_4_]^+^; found: 318.17390 C_15_H_28_O_4_NS [M+H]^+^, 340.15569 C_15_H_27_O_4_NNaS [M+Na]^+^, 335.20083 C_15_H_31_O_4_N_2_S [M+NH_4_]^+^; ***R*_f_:** 0.69 (DCM/MeOH 9:1, KMnO_4_); yield: 23.00 g; 78% (20.0 g scale, 92.48 mmol).

***tert*-Butyl 2-(((2-acetamidoethyl)thio)carbonyl)octanoate (6d): ^1^HNMR:** (400 MHz, CDCl_3_-d_1_) δ=0.86–0.89 (t, *J*=6.9 Hz, 3H), 1.27–1.29 (m, 8H), 1.46 (s, 9H), 1.85–1.88 (m, 2H), 1.96 (s, 3H), 3.00–3.13 (m, 2H), 3.37–3.51 (m, 3H), 5.85 (bs, 1H); **^13^C NMR:** (101 MHz, CDCl_3_-d_1_) δ=14.2, 22.7, 23.3, 27.3, 28.03, 28.06, 28.8, 29.03, 29.6, 31.6, 39.7, 61.3, 82.4, 168.0, 170.6, 196.3; **HRMS:** calc: 346.20466 C_17_H_32_O_4_NS [M+H]^+^, 368.18660 C_17_H_31_O_4_NNaS [M+Na]^+^, 363.23120 C_17_H_35_O_4_N_2_S [M+NH_4_]^+^; found: 346.20494 C_17_H_32_O_4_NS [M+H]^+^, 368.18686 C_17_H_31_O_4_NNaS [M+Na]^+^, 363.23182 C_17_H_35_O_4_N_2_S [M+NH_4_]^+^; ***R*_f_**: 0.79 (DCM/MeOH 9:1, KMnO_4_); yield: 9.61 g; 53% (12.77 g scale; 52.27 mmol).

***tert*-Butyl 3-((2-acetamidoethyl)thio)-3-oxo-2-phenylpropanoate (7d): ^1^H NMR:** (400 MHz, CDCl_3_-d_1_) δ=1.45 (s, 9H), 1.90 (s, 3H), 3.00–3.06 (m, 2H), 3.39–3.44 (m, 2H), 4.72 (s, 1H),5.83 (bs, 1H, NH), 7.35–7.41 (m, 5H); **^13^C NMR:** (101 MHz, CDCl_3_-d_1_) δ=23.1, 27.9, 29.3, 39.5, 66.4, 83.0, 128.6, 128.8, 128.9, 129.5, 132.6, 166.8, 170.6, 195.0; **HRMS:** calc.: 338.14206 C_17_H_24_O_4_NS[M+H]^+^, 360.12400 C_17_H_23_O_4_NNaS [M+Na]^+^, 355.16860 C_17_H_27_O_4_N_2_S [M+NH_4_]^+^; found: 338.14223 C_17_H_24_O_4_NS[M+H]^+^, 360.12417 C_17_H_23_O_4_NNaS [M+Na]^+^, 355.16909 C_17_H_27_O_4_N_2_S [M+NH_4_]^+^; ***R*_f_:** 0.56 (MeOH/DCM 9:1, KMnO_4_); yield: 1.60 g; 38% (2.9 g scale, 12.3 mmol).

**General procedure for the deprotection of compounds 1–7:**

The thioester was dissolved in abs. DCM (10 ml/100 mg) under argon. At 0 °C 2.5 eq. TiCl_4_ was dropwise added. The dark brown reaction mixture was stirred for 5 min at 0 °C, then for another 6 h at room temperature. After 6 h (DC-control) the reaction mixture was quenched with aq. Na_2_CO_3_-solution (10.0 eq. Na_2_CO_3_) in an ice bath to reach a final concentration of 0.1 M of product. The white suspension was filtered and washed twice with 10 ml MeOH. The combined solvents were evaporated at 30 °C under reduced pressure. The resulting brown solution or white slurry was transferred to polypropylene tubes and cooled for 2 h at −20 °C; after warming to 4 °C, Na_2_CO_3_ precipitated. The precipitate was removed by centrifugation at 4 °C/4000 rpm for 10 min. Subsequently, the supernatant was freeze dried. The resulting white/yellow solidwas transferred into polypropylene tubes and dissolved in SM16 medium to yield a 100 mM solution. The resulting slightly brown solution was centrifuged at 4 °C/4000 rpm for 10 min and the supernatant was sterile filtered and used directly for feeding experiments.

For analysis of the reaction product by NMR, the product was dissolved in D_2_O instead of SM3 medium.

**2-(((2-acetamidoethyl)thio)carbonyl)pent-4-enoic acid (1): ^1^H NMR:** (400 MHz, D_2_O-d_2_) δ=2.26 (s, 3H), 2.83–2.87 (m, 2H), 3.30–3.43 (m, 2H), 3.59–3.69 (m, 2H), 3.96–3.99 (t, *J*=7.6 Hz, 1H), 5.35–5.39 (m, 2H), 6.05–6.13 (m, 1H,); **^13^C NMR:** (101 MHz, D_2_O-d_2_) δ=22.8, 28.7, 34.2, 39.1,117.6, 135.6, 174.8, 175.7, 201.2; **HRMS:** calc.: 246.07946 C_10_H_16_O_4_NS[M+H]^+^, 268.06140 C_10_H_15_O_4_NNaS [M+Na]^+^; found: 246.07949 C_10_H_16_O_4_NS [M+H]^+^, 268.06048 C_10_H_15_O_4_NNaS [M+Na]^+^; ***R*_f_**: 0.12 (DCM/MeOH 9:1, KMnO_4_).

**2-(((2-acetamidoethyl)thio)carbonyl)pent-4-yn acid (2): ^1^H NMR:** (400 MHz, D_2_O-d_2_) δ=1.88 (s, 3H); 2.30–2.32 (t, *J*=2.6 Hz, 1H), 2.61–2.63 (m, 2H); 2.99–3.04 (m, 2H); 3.26–3.32 (m, 2H), 3.68-3.72 (t, *J*=7.6 Hz, 1H); **^13^C NMR:** (101 MHz, D_2_O-d_2_) δ=19.1, 22.5, 28.6, 38.9, 174.2, 174.7, 199.6; **HRMS:** cal.: 244.06381 C_10_H_14_O_4_NS [M+H]^+^; found: 244.06402 C_10_H_14_O_4_NS [M+H]^+^; ***R*_f_**: 0.18 (DCM/MeOH 1:9, KMnO_4_).

**2-(((2-acetamidoethyl)thio)carbonyl)butanoicacid (3): ^1^H NMR:** (400 MHz, D_2_O-d_2_) δ=1.13-1.17 (t, *J*=7.4 Hz, 3H), 2.04–2.11 (q, *J*=7.4 Hz, 2H), 2.23 (s, 3H), 3.27–3.40 (m, 2H), 3.59–3.69 (m, 2H), 3.74–3.77 (t, *J*=7.6 Hz, 1H); **^13^C NMR:** (101 MHz, D_2_O-d_2_) δ=12.0, 22.7, 23.9, 28.7, 39.1, 66.2, 174.7, 176.5, 201.92; **HRMS:** cal.: 234.07946 C_9_H_16_O_4_NS[M+H]^+^, 256.06140 C_9_H_15_O_4_NNaS [M+Na]^+^; found: 234.07957 C_9_H_16_O_4_NS[M+H]^+^, 256.06095 C_9_H_15_O_4_NNaS [M+Na]^+^; ***R*_f_**: 0.23 (DCM/MeOH: 9:1, KMnO_4_).

**2-(((2-acetamidoethyl)thio)carbonyl)-3-methylbutanoic acid (4): ^1^H NMR:** (400 MHz, MeOD-d_4_/D_2_O-d_2_) δ=0.86–0.87 (d, *J*=6.6 Hz, 3H), 0.92–0.94 (d, *J*=6.7 Hz, 3H), 1.95 (s, 3H), 2.25–2.34 (m, 1H), 3.00–3.04 (m, 2H), 3.18–3.21 (d, *J*=10.4 Hz, 1H), 3.18–3.32 (m, 2H); **^13^C NMR:** (101 MHz, MeOD-d_4_) δ=20.8, 21.3, 22.6, 29.2, 31.2, 39.9, 74.5, 173.7, 174.7, 199.7; **HRMS:** calc.: 248.09511 C_10_H_18_O_4_NS [M+H]^+^, 270.07705 C_10_H_17_O_4_NNaS [M+Na]^+^; found: 248.09535 C_10_H_18_O_4_NS [M+H]^+^, 270.07709 C_10_H_17_O_4_NNaS [M+Na]^+^; ***R*_f_**: 0.18 (DCM/MeOH 9:1, KMnO_4_).

**2-(((2-acetamidoethyl)thio)carbonyl)hexanoicacid (5): ^1^H NMR:** (400 MHz, CDCl_3_-d_1_) δ=1.12–1.16 (t, *J*=7.1 Hz, 3H), 1.53–1.57 (m, 4H), 2.05–2.11 (m, 2H), 2.25 (s, 3H) 3.28–3.43 (m, 2H), 3.63–3.67 (m, 2H), 3.82–3.85 (t, *J*=7.6 Hz, 1H); **^13^C NMR:** (101 MHz, CDCl_3_-d_1_) δ=16.5, 24.9, 25.4, 31.3, 32.1, 32.7, 41.7, 67.1, 177.2, 179.1, 204.6; **HRMS:** cal.: 262.11076 C_11_H_20_O_4_NS [M+H]^+^, 284.09270 C_11_H_19_O_4_NNaS [M+Na]^+^; found: 262.11083 C_11_H_20_O_4_NS [M+H]^+^, 284.09226 C_11_H_19_O_4_NNaS [M+Na]^+^; ***R*_f_:** 0.13 (DCM/MeOH 9:1, KMnO_4_).

**2-(((2-acetamidoethyl)thio)carbonyl)octanoicacid (6): ^1^H NMR:** (400 MHz, D_2_O-d_2_/MeOD-d_4_) δ=0.83-0.86 (m, 3H), 1.25–1.27 (m, 8H), 1.78–1.84 (m, 2H), 1.88 (s, 3H,), 2.94–2.98 (m, 2H), 3.26–3.27 (m, 2H), 3.41–3.44 (t, *J*=7.4 Hz, 1H); **^13^C NMR:** (101 MHz, MeOD-d_4_) δ=14.4, 22.6, 23.6, 28.8, 29.2, 30.2, 31.7, 32.8, 40.1, 65.9, 173.4, 175.6, 199.8; **HRMS:** calc.: 290.14206 C_13_H_24_O_4_NS [M+H]^+^, 312.12400 C_13_H_23_O_4_NaS [M+Na]^+^; found: 290.14226 C_13_H_24_O_4_NS [M+H]^+^, 312.12417 C_13_H_23_O_4_NaS [M+Na]^+^; ***R*_f_:** 0.23 (DCM/MeOH 9:1, KMnO_4_).

**3-((2-Acetamidoethyl)thio)-3-oxo-2-phenylpropanoic acid (7): ^1^H NMR:** (400 MHz, MeOD-d_4_) δ=1.88 (s, 3H), 2.58–2.61 (m, 2H), 2.98–3.01 (m, 2H), 3.35 (s, 1H), 7.24–7.33 (m, 5H); **HRMS:** calc.: 282.07946 C_13_H_16_O_4_NS[M+H]^+^; found: 282.07936 C_13_H_16_O_4_NS[M+H]^+^; ***R*_f_**: 0.2 (DCM/MeOH: 9:1, KMnO_4_).

**Mutagenesis of DEBS3 for an enzyme-directed mutasynthesisin *S. erythraea***

*S. erythraea* NRRL-B-24071, *S. erythraea*ΔAT6hyg^R^ [1] and *S. erythraea* AT6* were used for fermentation.

The alterations of the selected residues in the YASH motif [1] were accomplished by oligonucleotide-mediated mutagenesis and overlap-extension PCR using the Phusion Flash Master Mix (Thermo Fisher). Briefly, mutagenesis was achieved by performing PCR with designed oligonucleotide primers (Table 1) that include the desired mutation in their sequence (oligonucleotides 3 and 4) and flanking oligonucleotides (1 and 2) in a Piko™ Thermocycler with the following program: 3 min denaturation at 99 °C, 5 cycles of 15 s at 99 °C, annealing for 15 s at 65 °C and 40 s extension at 72 °C, 25 cycles of 15 s 99 °C, 40 s at 72 °C, and a final extension of 60 s at 72 °C. The *EcoR*V digested plasmid pKSSU89 was used as template [1].The PCR products were *Dpn*I digested, purified and precipitated using SureClean (Bioline, German) and redissolved in water. The two overlapping fragments were fused together in a subsequent extension reaction. The inclusion of flanking primers 1 and 2 in the extension reaction allowed the amplification of the fused product by PCR: 3 min at 99 °C, 25 cycles of 15 s at 99 °C and 40 s of 72 °C, 60 s of 72 °C. The final PCR products were gel-purified and cloned into *Sca*I linerarized pKSSU96 via SLIC-MIX [7]. Insert-containing clones were identified by colony PCR and analysis of isolated plasmids. Identity of the plasmids was confirmed by DNA sequencing. The DEBS3-encoding plasmids carrying the desired mutations were transformed into *E. coli* ET12567/pUZ8002 and then conjugated into *S. erythraea* ΔAT6hyg^R^. Conjugation and propagation of resulting clones was performed as in reference [8].

## Figures and Tables

**Table 1:** Oligonucleotides used in this study.

No.	Sequence
**1**	TTACGGCAAGTCGCGCGGGTCGTCGGGCCCGGTGCTGCTGGGTTCGGTG
**2**	AAGCCGCCTTCCAGGTCCACCGGCGTGGTGGCCAGCGGTCGCCAGTCG
**3**	CCAAGACGCTCCCGGCCGACGGCGCCGGCCACTCCCGCCACGTCGAGGAG
**4**	CTCCTCGACGTGGCGGGAGTGGCCGGCGCCGTCGGCCGGGAGCGTCTTGG

## Footnotes

Appendix A Supplementary data associated with this article can be found in the online version at doi:10.1016/j.dib.2015.09.052.
