# Supplementary material for: Data in support of substrate flexibility of a mutated acyltransferase domain and implications for polyketide biosynthesis
Source: Data Brief. 2015 Oct 14;5:528–36. doi: 10.1016/j.dib.2015.09.052 (PMC4625040; doi:10.1016/j.dib.2015.09.052)
Supplement: Supplementary file 1 — Supplementary material [file mmc1.docx]

**Supplementary File: NMR-spectra of the synthetic compounds**

**Title: Data in support of Substrate Flexibility of a Mutated Acyltransferase Domain and Implications for Polyketide Biosynthesis**

Authors: Stephan Klopries^§^, Kenny Bravo-Rodriguez^φ^, Kyra R. M. Koopmans^§^, Uschi Sundermann^$^, Samir Yahiaoui^&^, Julia Arens^§^, Susanna Kushnir^§^, Elsa Sanchez-Garcia^φ*^ and Frank Schulz^§*^

**Affiliations:**

^§^Fakultät für Chemie und Biochemie, Organische Chemie 1, Ruhr-Universität Bochum, Universitätsstraße 150, 44780 Bochum, Germany

^φ^ Max-Planck-Institut für Kohlenforschung, Kaiser-Wilhelm-Platz 1, 45470 Mülheim an der Ruhr, Germany

*corresponding authors

^$^Dr. Fooke-Achterrath Laboratorien GmbH, Habichtweg 16, 41468 Neuss, Germany

^&^Université de Caen Basse-Normandie, Centre d’Etudes et de Recherche sur le Médicament de Normandie, F-14032 Caen, France

**Contact email:** frank.schulz@rub.de

SI Figure 1: 1HNMR- and 13C-spectra of SNAC in CDCl3-d1.

**NMR-spectra of the malonic acid derivatives (1a, 2a+ 7a)**

SI Figure 2: ^1^H-NMR and ^13^C-spectra of 2-Allyl-malonic acid (**1a**) in MeOD-d_4_


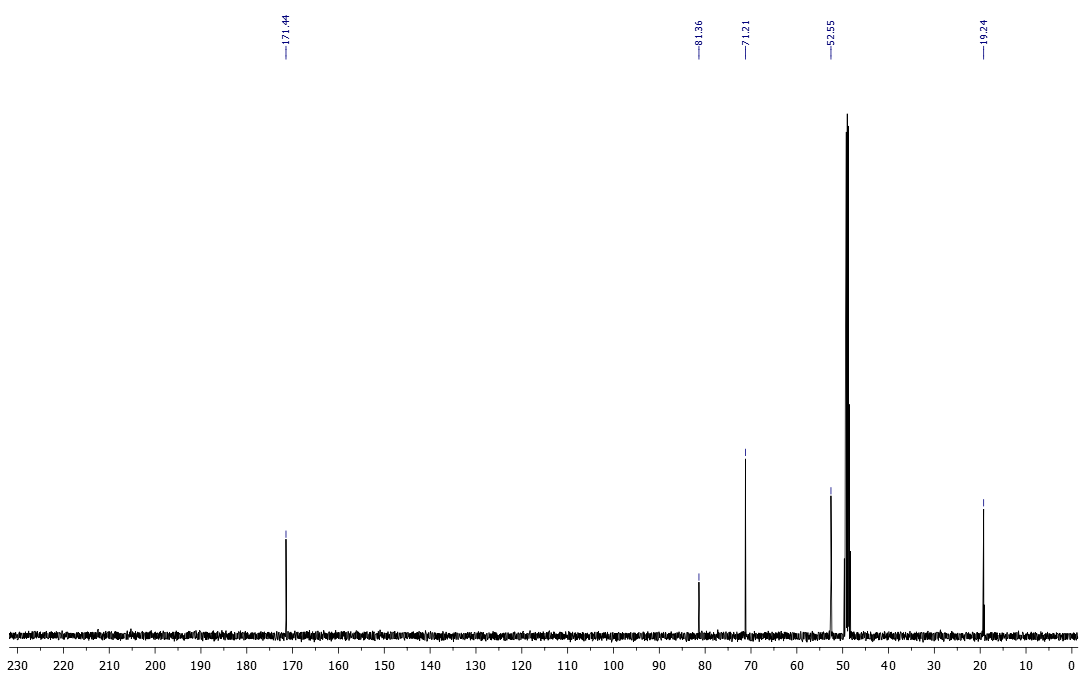

SI Figure 3: ^1^H-NMR and ^13^C-spectra of 2-(Prop-2-yn-1-yl)malonic acid (2a) in MeOD-d_4_


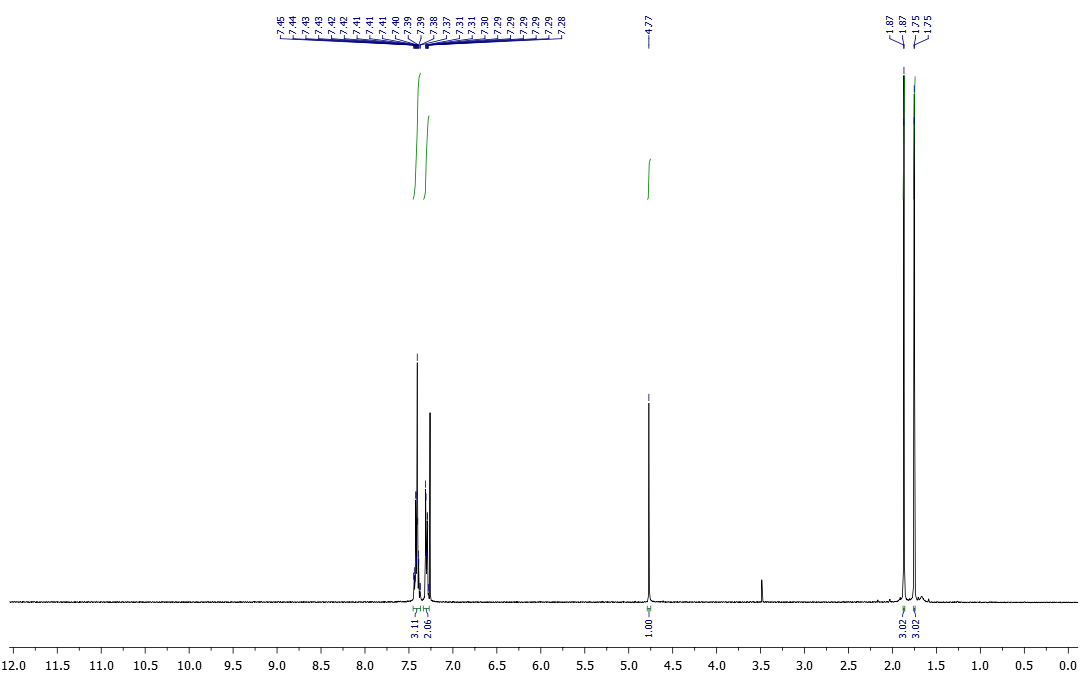

SI Figure 4: ^1^H-NMR and ^13^C-spectra of 2-Phenylmalonic acid (7a) in CDCl3-d1

**NMR-spectra of Meldrum’s acid derivatives 1b, 2b + 7b:**

SI Figure 5: ^1^H-NMR and ^13^C-spectra of 5-Allyl-2,2-dimethyl-1,3-dioxane-4,6-dione(1b) in CDCl_3_-d_1_

SI Figure 6:^1^H-NMR and ^13^C-spectra of 2,2-Dimethyl-5-(prop-2-yn-1-yl)-1,3-dioxane-4,6-dione (2b) in CDCl_3_-d_1_

SI Figure 7:^1^H-NMR- and ^13^C-spectra of 2,2-dimethyl-5-phenyl-1,3-dioxane-4,6-dione(7b) in CDCl_3_-d_1_

**NMR-spectra for the reductive alkylation of Meldrum’s acid 3b-6b:**


SI Figure 8:^1^H-NMR- and ^13^C-spectra of 5-isopropyl-2,2-dimethyl-1,3-dioxane-4,6-dione (4b) in CDCl_3_-d_1_

SI Figure 9:^1^HNMR- and ^13^C-spectra of 5-Ethyl-2,2-dimethyl-1,3-dioxane-4,6-dione (3b) in CDCl_3_-d_1_

SI Figure 10:^1^H-NMR and ^13^C-spectra of 5-Butyl-2,2-dimethyl-1,3-dioxane-4,6-dione (5b) in CDCl_3_-d_1_

SI Figure 11:^1^H-NMR and ^13^C-spectra of 5-Hexyl-2,2-dimethyl-1,3-dioxane-4,6-dione (6b) in CDCl_3_-d_1_

**NMR-spectra of *t*Butylmalonic acid 1c-7c:**

SI Figure 12:^1^H-NMR and ^13^C-spectra of 2-(tert-butoxycarbonyl)-3-methylbutanoic acid (4c) in CDCl_3_-d_1_

SI Figure 13:^1^H-NMR- and ^13^C-spectra of 2-(tert-butoxycarbonyl)pent-4-enoic acid (1c) in CDCl_3_-d_1_

SI Figure 14: ^1^H-NMR- and ^13^C-spectra of 2-(tert-butoxycarbonyl)butanoic acid (3c) in CDCl_3_-d_1_

SI Figure 15: ^1^H-NMR and ^13^C-spectra of 2-(tert-butoxycarbonyl)hexanoic acid (5c) in CDCl_3_-d_1_

SI Figure 16: ^1^H-NMR and ^13^C-spectra of 2-(tert-butoxycarbonyl)octanoic acid (6c) in CDCl_3_-d_1_

SI Figure 17:^1^H-NMR and ^13^C-spectra of 2-(^t^Butoxycarbonyl)-pent-4-yl acid (2c) in CDCl_3_-d_1_

SI Figure 18:^1^H-NMR and ^13^C-spectra of 3-(tert-butoxy)-3-oxo-2-phenylpropanoic acid (7c) in CDCl_3_-d_1_

**NMR-spectra of the Thioester 1d-7d:**

**SI Figure 19:**^1^H-NMR and ^13^C-spectra of tert-butyl 2-(((2-acetamidoethyl)thio)carbonyl)-3-methylbutanoate (**4d**) in CDCl_3_-d_1_

SI Figure 20:^1^H-NMR and ^13^C-spectra of tert-butyl 2-(((2-acetamidoethyl)thio)carbonyl)pent-4-enoate (1d) in CDCl_3_-d_1_

SI Figure 21:^1^H-NMR- and ^13^C-spectra of tert-butyl 2-(((2-acetamidoethyl)thio)carbonyl)butanoate (3d) in CDCl_3_-d_1_

SI Figure 22:^1^H-NMR and ^13^C-spectra of tert-butyl 2-(((2-acetamidoethyl)thio) carbonyl) hexanoate (5d) in CDCl_3_-d_1_

SI Figure 23:^1^H-NMR and ^13^C-spectra of tert-butyl 2-(((2-acetamidoethyl)thio)carbonyl)octanoate (6d) in CDCl_3_-d_1_

**SI Figure 24:**^1^HNMR- and ^13^C-spectra of *^t^*Butyl-2-(((2-Acetamidoethyl)thio)-carbonyl)pent-4-ynoate (**2d**) in CDCl_3_-d_1_

SI Figure 25: ^1^H-NMR and ^13^C-spectra of tert-butyl 3-((2-acetamidoethyl)thio)-3-oxo-2-phenylpropanoate (7d) in CDCl_3_-d_1_

**NMR-spectra of the Carboxylic acid1-7:**

SI Figure 26:^1^HNMR- and ^13^C-spectra of 2-(((2-acetamidoethyl)thio)carbonyl)-3-methylbutanoic acid (4) in D_2_O-d_2_ (1H-NMR), MeOD-d_4_ (^13^C-NMR)

SI Figure 27:^1^H-NMR- and ^13^C-spectra of 2-(((2-acetamidoethyl)thio)carbonyl)pent-4-enoic acid (1) in D_2_O-d_2_

SI Figure 28:^1^H-NMR and ^13^C-spectra of 2-(((2-acetamidoethyl)thio)carbonyl)butanoic acid (3) in D_2_O-d_2_

SI Figure 29:^1^H-NMR and ^13^C-spectra of  2-(((2-acetamidoethyl)thio)carbonyl)hexanoic acid (5) in D_2_O-d_2_

SI Figure 30:^1^H-NMR- and ^13^C-spectra of  2-(((2-acetamidoethyl)thio)carbonyl)octanoic acid (6) in D_2_O-d_2_ (^1^H-NMR), MeOD-d_4_ (^13^C-NMR)

SI Figure 31:^1^H-NMR and ^13^C-spectra of 2-(((2-acetamidoethyl)thio)carbonyl)pent-4-ynoic acid (2) in D_2_O-d_2_

SI Figure 32:^1^H-NMR and ^13^C-spectra of  3-((2-acetamidoethyl)thio)-3-oxo-2-phenylpropanoic acid(7) in MeOD-d_4_
