# Supplementary material for: Data in support of substrate flexibility of a mutated acyltransferase domain and implications for polyketide biosynthesis
Source: Data Brief. 2015 Oct 14;5:528–36. doi: 10.1016/j.dib.2015.09.052 (PMC4625040; doi:10.1016/j.dib.2015.09.052)
Supplement: Supplementary file 2 — Supplementary material [file mmc2.docx]

x
